# Reversible MR Findings in Marchiafava-Bignami Disease 

**DOI:** 10.1155/2019/1951030

**Published:** 2019-02-06

**Authors:** Carmine Franco Muccio, Luca De Lipsis, Rossella Belmonte, Alfonso Cerase

**Affiliations:** ^1^Neuroradiology Unit, Neuroscience Department, “G. Rummo” Hospital, Benevento, Italy; ^2^Anesthesia Department, “Sacred Heart of Jesus” Hospital, Benevento, Italy; ^3^Neuroimaging and Neurointervention Unit, NHS and University Hospital “Saint Mary alle Scotte”, Siena, Italy

## Abstract

Marchiafava-Bignami Disease (MBD) is a toxic demyelinating disease often diagnosed in chronic alcoholics. The disease process typically involves the corpus callosum and clinically presents with various manifestations resulting in MBD type A and type B on the basis of clinical condition, extent of callosal involvement and extracallosal involvement at brain magnetic resonance imaging (MRI), and prognosis. The death rate is high. We report a patient affected by MBD type B, who presented an isolated reversible splenial lesion at brain MRI and achieved a favorable recovery.

## 1. Introduction

Marchiafava-Bignami disease (MBD) is a rare neurological disease characterized by primary degeneration of the corpus callosum associated with chronic consumption of ethanol. MBD occasionally occurs in chronically malnourished, despite nonalcoholics. Clinical manifestation of MBD is nonspecific with a wide variation, including split-brain syndrome, difficult walking, para- or tetraparesis, altered mental status, seizure, and even coma or death. A deficiency of group B vitamins is the main etiopathogenic hypothesis, and many patients improve after the administration of these compounds. Magnetic resonance imaging (MRI) is generally used to support the diagnosis.

## 2. Case Presentation

**Figure 1:**
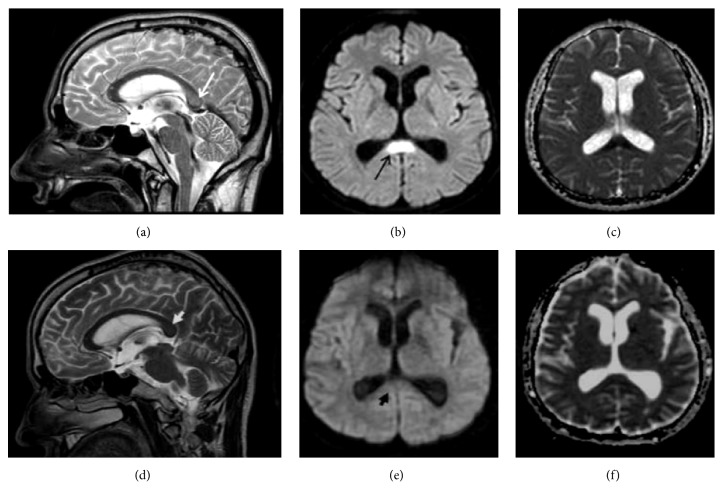
MRI studies at onset (a-c) and 1-month follow-up (d-f). Sagittal FSE-T2 image (a) demonstrating hyperintense regions in the splenium of the corpus callosum (*white arrow*). DWI (b) image shows its hyperintense signal (*black arrow*) and ADC map (c) shows its reduced diffusivity. Corresponding sagittal FSE-T2 image (d) and DWI images (e) showing normal signal in the splenium of the corpus callosum (*white arrow head *and* black arrow head*, respectively) where the ADC map (f) shows normalization of water diffusivity.

A 51-year-old man was admitted to the Emergency Room in a state of altered sensorium, with motor deficit of the lower limbs. The patient had been suffering from chronic alcohol abuse for 30 years, with an average daily intake of 700 mL/die. Physical examination revealed a Glasgow Coma Scale score of 9 (E2V3M4). No meningeal signs were present. Pupils showed normal size and were reactive to light. The patient did not suffer from diabetes, hypertension, seizure nor other significant diseases. Routine blood test and cerebrospinal fluid studies were negative. Electroencephalographic examination was normal. Brain MRI showed an area of high signal on fast-spin-echo (FSE) T2-weighted images and high signal on diffusion weighted imaging (DWI) with a decreased apparent diffusion coefficient (ADC) value of 670 x10-3 mm^2^/sec, observed with a region of interest size of 19 mm^2^, in the splenium of the corpus callosum (Figures 1(a)–1(c)). On the basis of history, findings on physical examination, and MR imaging features, the diagnosis of MBD was hypothesized. He was transferred to the intensive care unit where he required noninvasive ventilation and was treated with thiamine 400 mg/day [1] hydration and parenteral nutrition with vitamin supplement, so the electrolyte balance was quickly restored. We did not use steroid therapy. He showed improvement of symptoms with a good recovery in twenty days. Thirty-day follow-up brain MRI showed resolution of the abnormal callosal finding on both T2-weighted images and DWI-ADC maps (Figures 1(d)–1(f)).

## 3. Discussion

MBD is a rare disorder of unknown etiology characterized by demyelination of the corpus callosum with various clinical manifestations which is often mismanaged and mistreated [2]. It mainly affects the male population, in the age group between 40 and 60 years. Chronic alcohol abuse plays an important role in its development, even though MBD has been occasionally diagnosed in nonalcoholic patients as well [3]. These cases might have been diagnosed as a mild encephalitis/encephalopathy with a reversible splenial lesion, a recently described condition semiotically similar to MBD for the specific callosal localization, restricted water diffusivity, and reversibility at MRI [4].

Differential diagnosis includes other conditions which can present as splenial hyperintensity on T2/FLAIR like epilepsy, antiepileptic drug withdrawal, acute disseminated encephalomyelitis, infarction, viral and bacterial infections, especially influenza, HIV, and hypoglycaemia, other demyelinating diseases [5]. These conditions can be differentiated on the basis of clinical and laboratory analyses [6], such as occurred in the patient reported herein. Wernicke's encephalopathy was ruled out on the basis of clinical presentation and site of brain MRI lesion.

Despite several authors have postulated possible mechanism behind MBD, its pathophysiology still remains unclear. Nutritional deficiencies in newborns and in alcoholics and the use of some categories of drugs including benzodiazepines and antidepressants may be conditions predisposing the onset of the disease. Some authors believe to a genetic predisposition. Possible mechanisms include cytotoxic edema, blood-brain barrier breakdown, demyelination, and necrosis. Pathologically, MBD is characterized by symmetrical demyelination and necrosis of the central part of the corpus callosum, with relative sparing of thin upper and lower edges. Subsequently, necrosis leads to cavitation and atrophy of the corpus callosum in chronic stages [7]. Occasionally, similar lesions can involve other areas out of corpus callosum, such as anterior and posterior commissures, brachium pontis, optic chiasm, putamen, and frontal cortex [8, 9]. Few reports of partial damage to the corpus callosum and associated cortical lesions with good outcome are described.

Notably, a clinical and neuroradiological classification describes two subtypes of MBD.

(i) Type A is characterized by acute to subacute onset of altered state of level or state of consciousness, cognitive deterioration and language deficiencies (dysarthria, aphasia, etc.), hypertonia of the limbs, focal neurological deficits seizures, and pyramidal tract signs including hemiparesis or hemihypoesthesia, edema, and swelling of the entire corpus callosum at MRI, frequently with extracallosal lesions, resulting in an unfavourable and poorer prognosis.

(ii) Type B is characterized by an insidious clinical onset and progression, resulting in normal or slightly impaired level of consciousness, gait disturbance, dysarthria, signs of interhemispheric disconnection, high-signal intensity lesions on T2-weighted MR images partially involving the corpus callosum [10, 11], and less frequent extracallosal lesions associated with favorable prognosis including less severe and disabling clinical symptoms and less long-term disabilities.

Thus, diagnosis is made on the basis of clinical findings in combination with MRI features, such as that occurring in the patient reported herein. Therapy with thiamine and vitamine B complex, steroid, and antiepileptic is indicated [8]. A prompt therapy likely breaks the intramyelinic edema process and, subsequently, the disease progression [12, 13].

## 4. Conclusions

MBD diagnosis has been made possible as a result of recent advance in neuroimaging including DWI and ADC maps. Recognition of the neuroradiologic features is crucial in order to establish a proper diagnosis. Considering the complete reversibility of restricted diffusion in our patient, the lesion was probably the result of intramyelinic edema rather than cytotoxic edema or demyelination.
